# 5,7-Dihydroxyflavone Analogues May Regulate Lipopolysaccharide-Induced Inflammatory Responses by Suppressing I*κ*B*α*-Linked Akt and ERK5 Phosphorylation in RAW 264.7 Macrophages

**DOI:** 10.1155/2017/7898973

**Published:** 2017-04-30

**Authors:** Atsuyoshi Nishina, Kazue Shimizu, Mamoru Koketsu, Masayuki Ninomiya, Daisuke Sato, Takashi Suzuki, Satoshi Hayakawa, Hirokazu Kimura

**Affiliations:** ^1^College of Science and Technology, Nihon University, Chiyoda, Tokyo 101-0062, Japan; ^2^Department of Chemistry and Biomolecular Sciences, Faculty of Engineering, Gifu University, Gifu 501-1112, Japan; ^3^Department of Biomedical Information Engineering, Graduate School of Medical Science, Yamagata University, Yamagata 990-2332, Japan; ^4^School of Pharmacy, Nihon University, Funabashi, Chiba 274-8555, Japan; ^5^Nihon University School of Medicine, Itabashi, Tokyo 173-8610, Japan; ^6^National Institute of Infectious Diseases, Musashimurayama, Tokyo 208-0011, Japan

## Abstract

We studied the anti-inflammatory activity of twelve 5,7-dihydroxyflavone analogues in lipopolysaccharide- (LPS-) stimulated RAW 264.7 macrophages. We found that chrysin (**1**) and 4′-methoxytricetin (**9**) showed relatively significant anti-inflammatory activity and low cytotoxicity. Moreover,** 1** and** 9** recovered the expression levels of iNOS and COX2, as well as those of the intracellular inflammatory mediators IL-1*β* and IL-6, which were upregulated by LPS stimulation. In addition,** 1** and** 9** actively regulated the phosphorylation of I*κ*B*α*, leading to the activation of NF*κ*B. Phosphorylation of Akt and ERK5 (upstream of NF*κ*B) by LPS stimulation was significantly regulated by** 1** and** 9**, as well as by BIX 02189 and LY 294002, which are phosphorylation inhibitors of ERK5 and Akt, respectively. The results suggest that compounds** 1** and** 9** may suppress the levels of iNOS and COX2 by regulating phosphorylation of Akt, ERK5, and I*κ*B*α* and thus NF*κ*B-related signaling pathways, resulting in anti-inflammatory effects in the cells. Because** 1** and** 9** showed low cytotoxicity and regulated both PGE_2_ and NO production caused by inflammatory responses, they may hold promise as natural anti-inflammatory agents.

## 1. Introduction

Inflammatory responses constitute a biological defense system evoked by various biological events, including pathogenic infections. The major trigger is the recognition of pathogenic components by specific receptors [i.e., Toll-like receptors (TLRs)] of innate immunity, which play crucial roles in the initial promotion of signal transduction [1]. The main function of inflammatory responses is to exclude pathogens. However, responses can induce excessive cellular and tissue injuries due to overreaction by the host immunity, resulting in systemic inflammatory response syndrome. Therefore, the regulation of excessive inflammation is important for the maintenance of homeostasis [2].

The inflammatory responses arise from the humoral and cellular immunity systems [3]. Humoral immunity is associated with inflammatory cytokines (interleukin- (IL-) 1*β*, IL-6, and TNF-*α*) and other inflammatory mediators, such as prostaglandins [4], while cellular immunity consists of phagocytes such as neutrophils, monocytes, and macrophages. These components have additive and/or synergistic effects that can lead to excessive inflammation [5].

Many drugs have been developed to reduce excessive inflammation in vivo. Of these, steroidal or nonsteroidal anti-inflammatory drugs are generally used to eliminate acute inflammation. However, these drugs have a variety of side effects and are not suitable for the treatment of some chronic inflammatory diseases [6]. Therefore, it is important to develop other anti-inflammatory compounds with fewer side effects. Traditional medicines, such as Sino-Japanese medicines, frequently use plant extracts to treat chronic inflammatory diseases, including rheumatoid arthritis [7]. Previous studies suggest that these medicines contain active components, that is, polymethoxyflavones, which are not known to cause side effects [8].

Flavonoids are thought to be the active components of unrefined Chinese or Sino-Japanese traditional medicines used to provide anti-inflammatory effects. To date, more than 4000 flavonoids have been identified [9], and many of them are major components of the colors of flowers, fruit, and leaves [10]. Flavonoids occur as aglycones, glycosides, and methylated derivatives. The basic aglycone of flavonoids consists of a benzene ring (A) condensed with a six-membered ring (C) and a phenyl (B) group at the 2-position. Flavonoids can be classified into flavonols, flavones, catechins, flavanones, anthocyanidins, and isoflavones.

Since the anti-inflammatory effects of flavonoids are generally gentle, the mechanisms of action and structure-activity relationships have not been defined. However, many reports on the anti-inflammatory effects of isolated or synthesized flavonoids have been published. Indeed, many previous studies suggest that flavonoids have a variety of useful effects such as antitumor, antibacterial, antivirus, anti-inflammation, immunoregulation, and antithrombogenicity activities [11–15].

In the present study, we evaluated the anti-inflammatory effects of various 5,7-dihydroxyflavone analogues (12 compounds) on LPS-stimulated RAW 264.7 macrophages to confirm the anti-inflammatory structure-activity relationships of flavonoids [17].

## 2. Materials and Methods

### 2.1. Chemicals and Reagents

Lipopolysaccharide (LPS. coli serotype 026:B6) and N^G^-monomethyl-L-arginine (LN) were obtained from Sigma-Aldrich (St. Louis, MO, USA). NS-398 (NS) was purchased from Cayman Chemical (Ann Arbor, MI, USA). All other chemicals were reagent grade and purchased from Wako Pure Chemical (Osaka, Japan) unless otherwise stated.

### 2.2. Preparation of 5,7-Dihydroxyflavone Analogues

**Figure 1:**
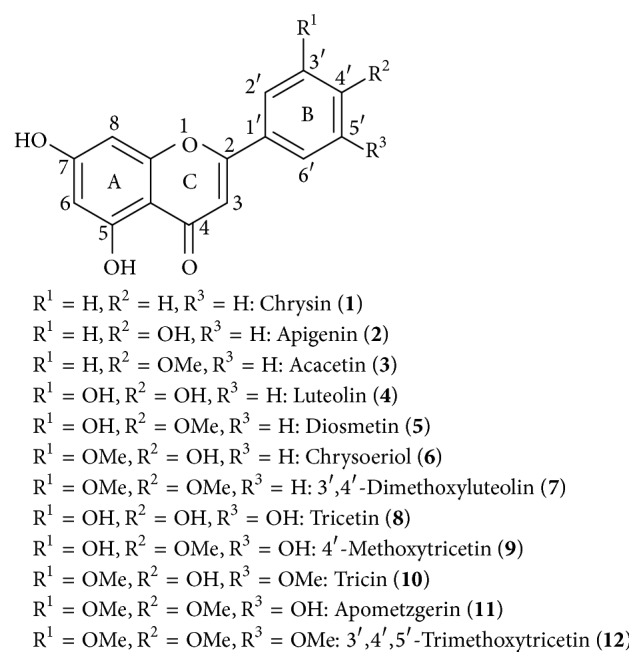
Chemical structures of 5,7-dihydroxyflavone analogues.

A series of 5,7-dihydroxyflavones (**2**,** 3**, and** 5**–**12**) was prepared by a convenient synthetic method as previously described [17]. To gain insight into the influence of hydroxylation and methylation in the B ring, we synthesized a series of 5,7-dihydroxyflavones through the condensation reactions of* tert*-butyldimethylsilyl- (TBS-) protected acetophenone and methyl benzoates, followed by acid cyclodehydration [18]. The condensation reaction of TBS-protected acetophenone and methyl benzoates was performed using 8 equiv. of lithium bis(trimethylsilyl)amide (LiHMDS) in THF at −78°C and with a temperature increase to room temperature for 1 day produced intermediates comprising mixtures of tautomers. These intermediates were subjected to acid cyclodehydration and deprotection with 0.5% H_2_SO_4_ in acetic acid at 100°C for 4.5 h. These reaction conditions resulted in the formation of 5,7-dihydroxyflavones, with the chemical structures depicted in Figure 1. Purity of all 5,7-dihydroxyflavones was 98% or more as results of measurement by HPLC.

### 2.3. Cell Culture

RAW 264.7 macrophages were purchased from Riken Cell Bank (Tsukuba, Ibaraki, Japan) and cultured in Dulbecco's modified Eagle's medium (DMEM) supplemented with 10% fetal bovine serum, 2 mM L-glutamine, 100 units /ml penicillin, and 0.1 mg /ml streptomycin. Cultures were maintained at 37°C in a 5% CO_2_ humidified atmosphere and experiments were conducted on cells at approximately 80–90% confluence [19].

### 2.4. Cell Toxicity Assay

RAW 264.7 cells were seeded into 96-well plate (10^4^ cells/well) with DMEM supplemented with 10% fetal bovine serum. Various concentrations of dimethyl sulfoxide (DMSO) solution of inhibitors and 5,7-dihydroxyflavone analogues or reference compounds were added followed by incubation for 2 days. Cytotoxicity by the modified MTT (3-(4,5-dimethylthiazol-2-yl)-2,5-diphenyltetrazolium bromide) method was measured using an available Kit [Cell Counting Kit-8 (Dojindo, Kumamoto, Japan)] according to the instruction by the manufacturer. Absorbance was measured at 450 nm by using Sunrise Absorbance Reader (Tecan, Männedorf, Switzerland) [20].

### 2.5. Nitric Oxide (NO) Production Analysis

RAW 264.7 cells were seeded in 10^4^ cells/well into a 96-well plate and incubated 2 hours. Then they were treated with an inhibitor of appropriate concentration for one hour followed by stimulation with LPS of 100 ng/well with or without various concentrations of a dimethyl sulfoxide (DMSO) solution of 5,7-dihydroxyflavone analogue or reference compounds for 16 hours. One hundred microliters of the culture supernatants were transferred into another 96-well plate and treated with 100 *μ*l of Griess reagent solution (a mixture of 0.05%  *N*-(1-naphthyl), ethylenediamine dihydrochloride, 0.5% sulfanilic acid, and 2.5% phosphoric acid). After 10 minutes at room temperature, absorbance was measured at 570 nm by using Sunrise Absorbance Reader (Tecan, Männedorf, Switzerland) [21].

### 2.6. Measurement of Prostaglandin E_2_ (PGE_2_)

RAW 264.7 cells were seeded in 10^4^ cells/well into a 96-well plate and incubated 2 hours. Then they were treated with an inhibitor followed by stimulation by LPS with or without a 5,7-dihydroxyflavone analogue at the same condition as above-mentioned. PGE_2_ measurements were conducted using a PGE_2_ EIA Kit (Enzo Life Sciences, Farmingdale, NY) according to the manufacturer's instructions with a microplate reader at 405 nm correction at 580 nm.

### 2.7. Detection of Proteins

RAW 264.7 cells were seeded into 6-well plate (10^6^ cells/well) with DMEM and supplemented with 10% fetal bovine serum. Various concentrations of dimethyl sulfoxide (DMSO) solution of inhibitors and 5,7-dihydroxyflavone analogues or reference compounds were added followed by incubation for 2 days; the 6-well plates were placed on ice and each well was washed with PBS and subsequently lysed with 150 *μ*L of 20 mM Tris-HCl buffer (pH 8.0), containing 150 mM NaCl, 2 mM EDTA, 1% Nonidet P-40 (w/v), 1% sodium deoxycholate (w/v), 0.1% sodium dodecyl sulfate (w/v), 50 mM NaF, 0.1% aprotinin (w/v), 0.1% leupeptin (w/v), 1 mM Na_3_VO_4_, and 1 mM phenylmethylsulphonylfluoride (PMSF). Cell lysates were collected by using a cell scraper and centrifuged at 15000 ×g for 30 min at 4°C. The supernatant was collected and the overall protein concentration was determined by a Protein Assay Reagent Kit (Cytoskeleton, Denver, CO) with BSA as the standard. Supernatant fluids containing proteins (20 *μ*g) were mixed with lithium dodecyl sulfate (LDS) sample buffer (Invitrogen Corp, Carlsbad, CA) and incubated for 5 min at 80°C. Proteins in the samples were separated on SDS-polyacrylamide gel electrophoresis, and the proteins in gels were electroblotted onto polyvinylidene fluoride (PVDF) filters (Hybond-P, 0.2 *μ*m; GE Healthcare, Little Chalfont, UK). Immunoblotting analysis was performed by using monoclonal antibodies against glyceraldehyde-3-phosphate dehydrogenase (GAPDH), induced nitric oxide synthase (iNOS), cyclooxygenase 1 (COX1), COX2, tumor necrosis factor-*α* (TNF-*α*), Interleukin-1*β* (IL-1*β*), IL-6, inhibitor of I*κ*B kinase (IKK), phospho-IKK, nuclear factor of kappa light polypeptide gene enhancer in B-cells inhibitor *α* (I*κ*B*α*), phospho-I*κ*B*α*, nuclear factor-kappa B (NF*κ*B), phospho-NF*κ*B, Akt, phospho-Akt, extracellular signal-regulated kinase (ERK)1/2, phospho-ERK1/2, c-jun N-terminal kinase (JNK), phospho-JNK, mitogen-activated protein kinase p38 (p38MAPK), phospho-p38MAPK, ERK5, and phospho-ERK5 (Cell Signaling Technology, Lake Placid, NY) as the primary antibodies, followed by reaction with horseradish peroxidase-conjugated anti-rabbit IgG antibodies from Sigma-Aldrich as the secondary antibody. Molecular sizes of the targeted proteins including GAPDH, iNOS, COX1, COX2, TNF-*α*, IL-1*β*, IKK, I*κ*B*α*, NF*κ*B, Akt, ERK1, ERK2, JNK1, JNK2, p38MAPK, and ERK5 were 37, 130, 70, 74, 25, 31, 85, 39, 65, 60, 44, 42, 54, 46, 40, and 115 kD, respectively. Primary and secondary antibodies were diluted 1000 and 3000 times for use, respectively. The blots were developed by the enhanced chemiluminescence method (Western Lightning ECL Pro; Perkin Elmer, Waltham, MA) [22]. In this study, we did not examine the negative control assays because the monoclonal antibodies used in this study showed high specificity [23].

### 2.8. Statistical Analysis

The results were expressed as mean ± standard deviation (SD). The significant differences between the groups compared were determined by Steel-Dwass test.

## 3. Results

### 3.1. Cytotoxicity and Downregulation of NO and PGE_2_ Productions by 5,7-Dihydroxyflavone Analogues and Inhibitors

**Figure 2:**
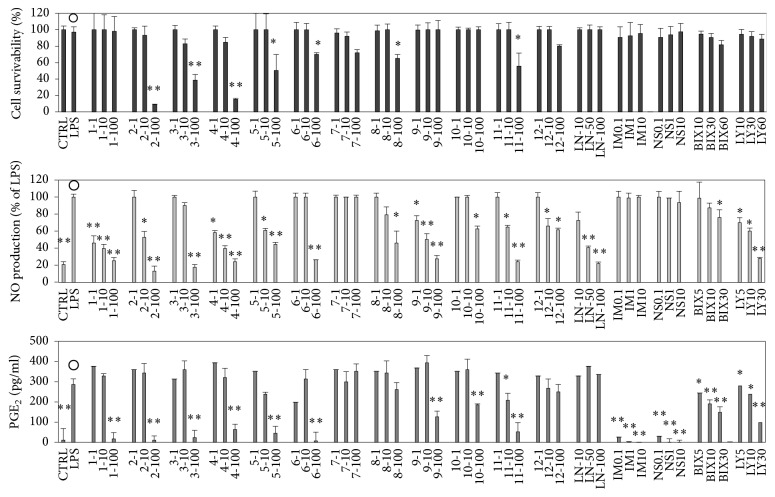
Cytotoxic effects and production inhibitions of nitric oxide (NO) and prostaglandin E_2_ (PGE_2_) by 5,7-dihydroxyflavone analogues (12 compounds) and five inhibitors in LPS-stimulated RAW 264.7 cells. RAW 264.7 cells were seeded into a 96-well plate (10^4^ cells/well) and incubated for 2 hours. Then they were stimulated with LPS of 100 ng/well with or without various concentrations of a dimethyl sulfoxide (DMSO) solution of a compound for 16 hours. Detailed procedures for cytotoxicity (A), NO (B), and PGE_2_ (C) were described in the text. Labels present the compound names and concentrations (*μ*M). Data are expressed as mean ± SD from 3 independent experiments. Control group was signified by the open circles. ^*∗*^Significance: *p* < 0.05. ^*∗∗*^Significance: *p* < 0.01. LPS: lipopolysaccharide; LN: NG-monomethyl-Larginine; IM: indomethacin; NS: NS-398; BIX: BIX 02189; and LY: LY294002.

We examined the effects of twelve 5,7-dihydroxyflavone analogues on cytotoxicity and the inhibition of nitric oxide (NO) and prostaglandin E_2_ (PGE_2_) production on LPS-stimulated RAW 264.7 cells (Figure 2). We also examined five inhibitors including NG-monomethyl-L-arginine (LN), indomethacin (IM), NS-398 (NS), BIX 02189 (BIX), and LY294002 (LY) as the reference compounds to compare the effects. Three of the 5,7-dihydroxyflavone analogues (**1**,** 9**, and** 10**) did not induce a significant amount of cell death even at 100 *μ*M. Compounds** 1**,** 4**, and** 9** inhibited LPS-induced NO production at comparatively low concentrations. In addition, the inhibition of LPS-induced PGE_2_ production by compounds** 7** and** 12** was relatively weak. On the other hand, the reference compounds, LN, IM, NS, BIX, and LY, did not show significant cytotoxic effects (Figure 2). Although the half maximal inhibitory concentration (IC_50_) of LN for NO was about 50 *μ*M, it did not inhibit the production of PGE_2_. On the contrary, two reference compounds, IM, a nonselective COX inhibitor, and NS, a specific inhibitor of COX2, downregulated the production of PGE_2_ but not NO. Moreover, BIX and LY inhibited LPS-induced production of NO and PGE_2_ in a dose-dependent manner. Relationships between chemical structures and biological activities could not be confirmed, but the cytotoxic effects of** 1** and** 9** were relatively low and the inhibitory effects on LPS-induced NO and PGE_2_ production by** 1** and** 9** were stronger than other 5,7-dihydroxyflavone analogues. Thus, compounds** 1** and** 9** were selected as the representatives from twelve kinds of 5,7-Dihydroxyflavone analogues and effects of compounds** 1** and** 9** in RAW 264.5 cells were measured in detail hereafter.

### 3.2. Effects of 1, 9, and Inhibitors on Expression of iNOS, COX1, and COX2

**Figure 3:**
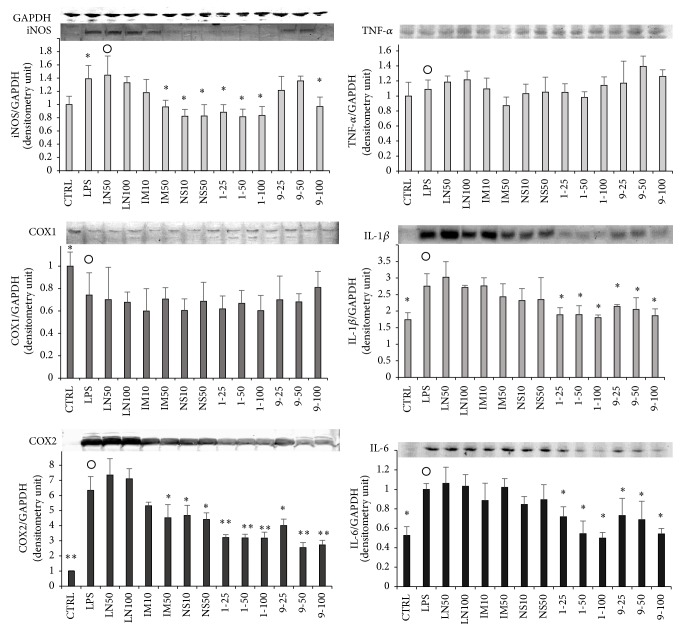
Effects of 1, 9, and inhibitors on expression levels of iNOS, COX1, COX2, and inflammation-related proteins in RAW 264.7 cells. RAW 264.7 cells were seeded into a 6-well plate (3  ×  10^5^ cells/well) and incubated for 2 hours. Then they were stimulated with LPS of 100 ng/well with or without various concentrations of a dimethyl sulfoxide (DMSO) solution of a 5,7-dihydroxyflavone analogue or an inhibitor for 16 hours. Detailed procedures for the protein collection/evaluation are described in the text. Same cell lysate was used for the assessment of six proteins and GAPDH was used as the standard for quantification of all proteins. Labels present the compound names and concentrations (*μ*M). Data are expressed as mean ± SD from 3 independent experiments. Control group were presented by the open circles. ^*∗*^Significance: *p* < 0.05 and ^*∗∗*^Significance: *p* < 0.01.

The effects of** 1**,** 9**, and three inhibitors (LN, IM, and NS) on the expression levels of iNOS, COX1, and COX2 are shown in Figure 3. The concentrations of iNOS and COX2 were significantly increased, while COX1 was decreased by LPS stimulation. The level of COX1 was not restored by the compounds (Figure 3). Moreover, the expression levels of iNOS and COX2 were not affected by the addition of LN. Levels of iNOS and COX2 were decreased by the addition of more than 50 *μ*M IM or NS, although these results were not significant. On the other hand, both iNOS and COX2 levels were decreased significantly by the addition of** 1** or** 9**.

### 3.3. Effects of 1, 9, and Inhibitors on Intracellular Levels of Inflammation-Related Proteins

We also examined the effects of** 1**,** 9**, and the inhibitors (LN, IM, and NS) on the expression of inflammatory mediators, TNF-*α*, IL-1*β*, and IL-6 (Figure 3). Intracellular levels of TNF-*α* were not affected by LPS stimulation or the addition of the compounds (Figure 3), while intracellular levels of IL-1*β* and IL-6 were increased by LPS stimulation. IM and NS restored the levels slightly, though these results were not significant; LN had no effect. On the other hand, the increased intracellular levels of IL-1*β* and IL-6 were significantly reduced by the addition of** 1** or** 9** (*p* < 0.05). It was presumed that the downregulation of LPS-induced NO and PGE_2_ was mainly caused by decreases in the expression levels of iNOS and/or COX2 (Figure 3).

### 3.4. Effects of 1, 9, and Inhibitors on NF*κ*B-Related Proteins

It is suggested that LPS activates nuclear factor-kappa B (NF*κ*B) through stimulation of TLR4, resulting in expression of iNOS, COXs, and other inflammatory-related cytokines [24]. Nuclear translocation of NF*κ*B, mainly in the cytoplasm, is inhibited by bonding with nuclear factor of kappa light polypeptide gene enhancer in B-cells inhibitor-*α* (I*κ*B*α*). LPS stimulation phosphorylates I*κ*B kinase (IKK), which is the enzyme complex that phosphorylates the serine residues of I*κ*B*α*. I*κ*B*α* is then phosphorylated by phosphorylated IKK, and, finally, NF*κ*B is activated by the degradation of phosphorylated I*κ*B*α* via the ubiquitin proteasome system. Activated and nuclear-translocated NF*κ*B induces the expression of iNOS, COXs, and other inflammatory cytokines, and inflammation progresses.

**Figure 4:**
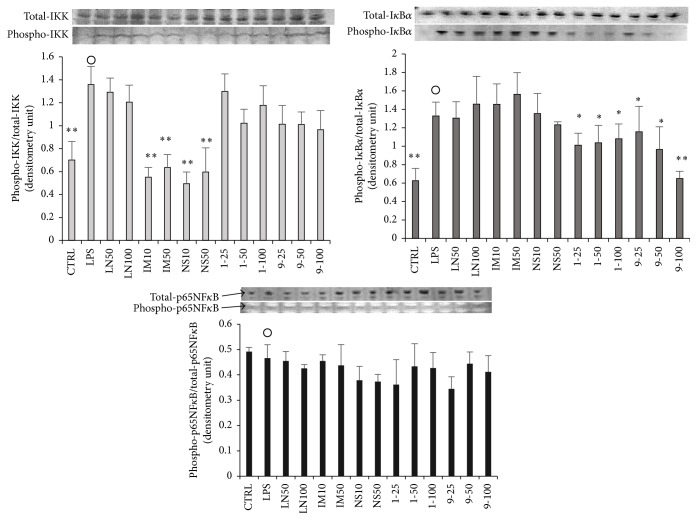
Effects of** 1**,** 9**, and inhibitors on NF*κ*B-related proteins in RAW264.7 cells. RAW264.7 cells were seeded into a 6-well plate (3 × 10^5^ cells/well) and incubated for 2 hours. Then they were stimulated with LPS of 100 ng/well with or without various concentration of a dimethyl sulfoxide (DMSO) solution of a 5,7-dihydroxyflavone analogue or an inhibitor for 15 min. Detailed procedures for the protein collection/evaluation are described in the text. Labels present the compound names and concentrations (*μ*M). Data are expressed as mean ± SD from 3 independent experiments. Control group were presented by open circles. ^*∗*^Significance: *p* < 0.05 and ^*∗∗*^Significance: *p* < 0.01.

The effects of** 1**,** 9**, and COX inhibitors on the expression levels and phosphorylation of IKK, I*κ*B*α*, and NF*κ*B were evaluated (Figure 4). First, the phosphorylation levels of IKK and I*κ*B*α* were significantly induced (*p* < 0.01) by LPS stimulation without 1, 9, or inhibitors, while NF*κ*B phosphorylation remained the same, although the expression levels of the three proteins were not changed by LPS stimulation. The phosphorylation of IKK was not affected by LN,** 1**, or** 9** but was significantly suppressed by IM and NS. On the other hand, the phosphorylation of I*κ*B*α* was not affected by LN, IM, or NS but was significantly suppressed by** 1** and** 9**. Moreover, the addition of the other compounds described in Figure 4 had no significant effect on the phosphorylation of NF*κ*B. From these results, it was deduced that** 1** and** 9** or IM and NS downregulated the inflammatory reactions due to reversal of I*κ*B*α* or IKK phosphorylation, respectively.

### 3.5. Effects of** 1**,** 9**, and Inhibitors on Intracellular Signal Transduction-Related Kinases

**Figure 5:**
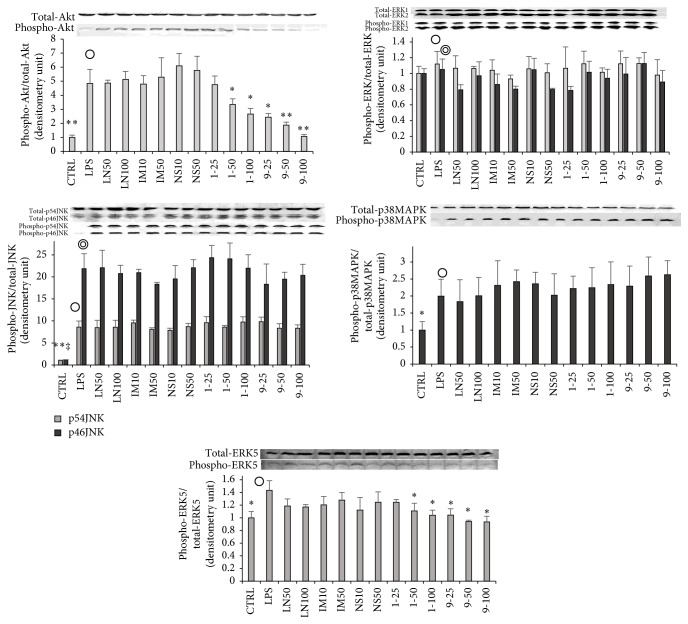
Effects of** 1**,** 9**, and inhibitors on signal-regulated kinases in RAW 264.7 cells. RAW 264.7 cells were seeded into a 6-well plate (3 × 10^5^ cells/well) and incubated for 2 hours. Then they were stimulated with LPS of 100 ng/well with or without various concentrations of a dimethyl sulfoxide (DMSO) solution of a 5,7-dihydroxyflavone analogue or an inhibitor for 15 min. Detailed procedures for the protein collection/evaluation are described in the text. Labels present the compounds and concentrations (*μ*M). Data are expressed as mean ± SD from 3 independent experiments. Control group were presented by the open circles. ^*∗*^Significance: *p* < 0.05 and ^*∗∗*^ and ^‡^Significance: *p* < 0.01.

Some studies on the interactions between inflammation and mitogen-activated protein kinase (MAPK), which exists in the cytoplasm, reported that the phosphorylation of both p38MAPK and Akt is associated with inflammatory reactions [25–28]. We examined the effects of compounds** 1** and** 9** and the inhibitors on the phosphorylation of MAPKs and Akt (Figure 5). LPS stimulation enhanced the phosphorylation of Akt, JNK, p38MAPK, and ERK5 but had no such effect on ERK1/2. The phosphorylation of intracellular signal transduction-related kinases was not influenced by LN, IM, or NS (Figure 5). Moreover, it was confirmed that** 1** and** 9** reversed the phosphorylation of Akt and ERK5 induced by LPS stimulation. From these results, it was deduced that inflammatory reactions may be depressed by** 1** or** 9** via reversal of the phosphorylation of Akt and ERK5 induced by LPS stimulation followed by downregulation of I*κ*B*α* phosphorylation.

### 3.6. Effects of Akt or ERK5 Inhibitor on LPS-Induced Inflammatory Reaction

**Figure 6:**
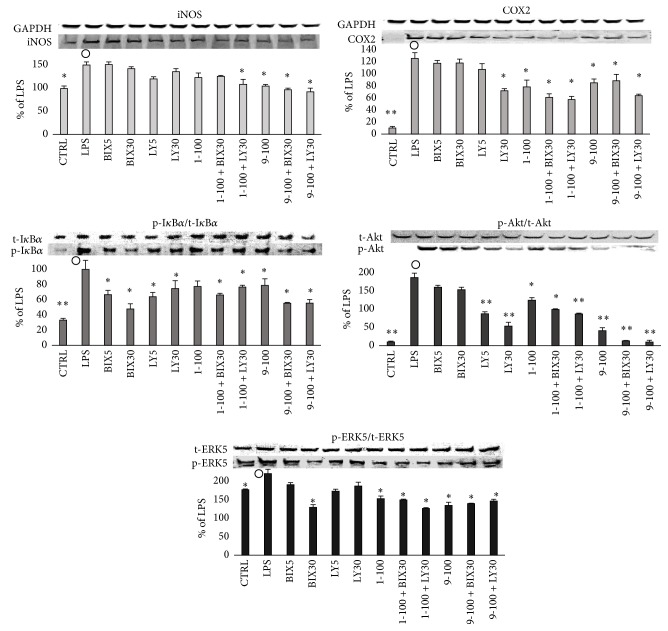
Effects of 1 or 9 with or without Akt or ERK5 inhibitor on inflammatory rerated proteins in RAW 264.7 cells. RAW 264.7 cells were seeded into a 6-well plate (3 × 10^5^ cells/well) and incubated for 2 hours. Then they were treated with an inhibitor of appropriate concentration for one hour followed by stimulation with LPS of 100 ng/well with or without various concentration of a dimethyl sulfoxide (DMSO) solution of a 5,7-dihydroxyflavone analogue for 15 min (I*κ*B*α*, Akt, and Erk5) or 16 hours (iNOS and COX2). Labels present the compound names and concentrations (*μ*M). Data are expressed as mean ± SD from 3 independent experiments. Control group were presented by the open circles. ^*∗*^Significance: *p* < 0.05 and ^*∗∗*^Significance: *p* < 0.01.

Though ERK5 has the TEY array, as well as classical ERK1/2 [29], it is not activated by MAPK kinase (MEK1/2) but is specifically activated by MEK5. Previous reports showed that ERK5 is activated by hyperosmosis or oxidative stress and it is recognized as a stress responder MAPK, similar to JNK and p38MAPK [30, 31]. However, because it was confirmed that ERK5 can be activated even by trophic factors, such as epidermal growth factor (EGF), nerve growth factor (NGF), and serum [32, 33], it is now recognized that it has multiple functions, including those involved in inflammation [29]. Thus, to confirm the anti-inflammatory mechanisms of compounds** 1** and** 9**, we examined the effects of LY294002 (LY), an Akt phosphorylation inhibitor, through the inhibition of PI3 kinase and BIX02188 (BIX), a specific inhibitor of ERK5 phosphorylation in LPS-stimulated RAW 264.7 cells. The results (Figure 2) showed that neither IM nor NS—COX inhibitors—inhibited the production of NO. However, they did downregulate the production of PGE_2_, resulting from LPS stimulation (Figure 2). On the other hand, LN—a competitive inhibitor of iNOS—regulated the production of NO, but not the production of PGE_2_. Compounds** 1** and** 9** as well as LY and BIX downregulated both NO and PGE_2_ production induced by LPS stimulation in a dose-dependent manner. Among the proteins described in Figures 3–5, the effects of BIX or LY on iNOS, COX2, IkB*α*, Akt, and ERK5, which were remarkably recovered by** 1** and** 9**, are shown in Figure 6. BIX and LY downregulated the expression of iNOS and COX2 but there was no significant downregulation of IkB*α*, Akt, or ERK5 phosphorylation induced by LPS stimulation. Moreover, it was suggested that the effects of** 1**,** 9**, BIX, or LY were similar because the tendency of reinforcement had been observed by using** 1** or** 9** with BIX or LY (Figure 6). Furthermore, because BIX or LY are inhibitors of ERK5 or Akt phosphorylation, respectively, it was assumed that the inhibition of ERK5 or Akt phosphorylation is necessary to achieve the anti-inflammatory effects of** 1** and** 9**.

## 4. Discussion

Many reports have shown the anti-inflammatory effects of the extracted and synthesized flavonoids [34]. However, their anti-inflammatory effects are mild [35]. In addition, the relationships between their chemical structures and biological activities are not fully understood. We studied the cytotoxicity and anti-inflammatory effects of twelve 5,7-dihydroxyflavone analogues in inflammatory cells (macrophages). We found that compounds** 1** and** 9** showed significant anti-inflammatory effects and low cytotoxicity. Moreover,** 1** and** 9** restored the expression levels of iNOS and COX2, as well as the intracellular inflammatory mediators, IL-1*β* and IL-6, which were upregulated by LPS stimulation.

**Figure 7:**
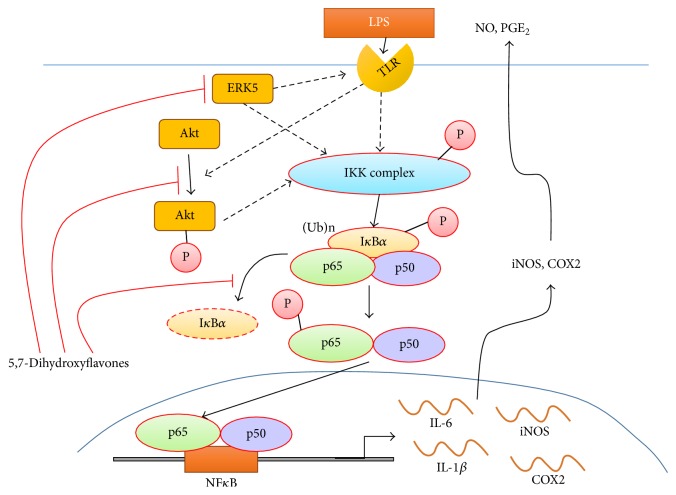
The suggested pathway of inflammatory reaction [16] and a tentative mechanism of anti-inflammation by 5,7-dihydroxyflavones. They significantly suppressed the phosphorylation of I*κ*B*α* by inhibition of both ERK5 and Akt phosphorylation and inhibition of NF*κ*B activity, resulting in the inhibition of iNOS and COX2 production. TLR: toll-like receptor, Ub: ubiquitin, and P: phosphoric acid.

It is suggested that the inflammatory response is composed of step-by-step reactions involving inflammatory cascades [36]. Of these cascades, inflammatory response-associated intracellular signaling pathways involving NF*κ*B may play crucial roles in the production of inflammatory cytokines in vivo [37]. The key molecule, NF*κ*B, is located downstream of the inflammatory cascades. Another molecule, I*κ*B*α*, binds to NF*κ*B and regulates NF*κ*B activity. Other important signaling pathways, such as those of Akt and ERK5, are located upstream of NF*κ*B and these molecules regulate the activation of NF*κ*B. Moreover, it is known that Akt regulates the expression of TNF-*α*, NF*κ*B, and AP-1 complex [38]. Thus, regulation of these signaling pathways is important in the prevention of excessive inflammatory responses. In the present study,** 1** and** 9** significantly suppressed the phosphorylation of Akt and ERK5 in LPS-stimulated macrophages. In addition, BIX and LY (specific inhibitors of ERK5 or Akt) showed similar effects to** 1** and** 9** in LPS-stimulated cells. Thus, it can be considered that compounds** 1** and** 9** significantly suppressed the phosphorylation of I*κ*B*α* by inhibition of both ERK5 and Akt phosphorylation and inhibition of NF*κ*B activity resulting in the inhibition of iNOS and COX2 production. A tentative mechanism of anti-inflammatory response by** 1** and** 9** via downregulation of phosphorylation of ERK5 or Akt was summarized in Figure 7. These processes may be associated with the suppression of inflammatory responses in LPS-stimulated macrophages.

Here, we used some specific inhibitors (LN, IM, and NS) as reference compounds. First, LN is a specific inhibitor of NOS due to competitive binding at the arginine binding site in the enzyme, resulting in inhibition of NO production [39]. Our results showed that LN affected NO production alone. Thus, the effects of the compound were compatible with earlier reports [40]. Second, IM is a nonspecific inhibitor of COXs, while NS selectively inhibits COX2. Thus, both compounds act as anti-inflammatory agents due to the inhibition of PGE_2_ production [41–43]. In this study, compounds** 1** and** 9** inhibited both NO and PGE_2_ production, while IM and NS inhibited PGE_2_ production alone (Figure 2). Moreover,** 1** and** 9** inhibited I*κ*B*α* phosphorylation, while IM and NS inhibited the phosphorylation of IKK (Figure 4). Thus, the mechanisms of the anti-inflammatory effects between our compounds and the reference compounds are distinct.

It is known that the flavonoid kaempferol inhibits inflammatory reactions via inhibition of Src, Syk, interleukin-1 receptor-associated kinase 1 (IRAK1), and IRAK4 activation, followed by inhibition of the transcriptional activity of NF*κ*B and AP-1 [38]. In addition, compound** 8** used in this study and PI3K/Akt inhibitor (LY294002) both inhibited the production of TNF-*α* [26]. Moreover, kolaviron (a tetramer of** 4**) inhibited inflammatory responses, including the production of IL-6, by inhibiting the activation of ERK1/2, NF*κ*B, p38MAPK, Akt, phospho-c-Jun, and JNK [44]. Among the 12 analogues, we selected** 1** and** 9** as candidate anti-inflammatory drugs, because they have low toxicity and strong anti-inflammatory effects on RAW 264 cell macrophages (Figure 2). The results showed that** 1** and** 9** effectively inhibited the phosphorylation of Akt, ERK5, and I*κ*B*α*, resulting in the suppression of iNOS and COX2 expression and the inhibition of NO and PGE_2_. The actions of** 1** and** 9** in cells under LPS stimulation were similar to those of Akt and ERK5 inhibitors. Thus, we suspect that the anti-inflammatory effects of** 1** and** 9** may be due to the suppression of Akt and/or ERK5 phosphorylation in cells. Further studies are needed to gain a better understanding of the anti-inflammatory mechanisms of flavonoids and relationship between Akt or ERK5 and the inflammatory responses.

## 5. Conclusion

We found that chrysin (**1**) and 4′-methoxytricetin (**9**) showed significant anti-inflammatory activity and low cytotoxicity. Moreover,** 1** and** 9** recovered the expression levels of iNOS and COX2, as well as those of the intracellular inflammatory mediators IL-1*β* and IL-6, which were upregulated by LPS stimulation. In addition,** 1** and** 9** actively regulated the phosphorylation of I*κ*B*α*, leading to the activation of NF*κ*B. Phosphorylation of Akt and ERK5 (upstream of NF*κ*B) by LPS stimulation was significantly regulated by** 1** and** 9**, as well as by BIX 02189 and LY 294002, which are phosphorylation inhibitors of ERK5 and Akt, respectively. The results suggest that compounds** 1** and** 9** may suppress the levels of iNOS and COX2 by regulating phosphorylation of Akt, ERK5, and I*κ*B*α* and thus NF*κ*B-related signaling pathways, resulting in anti-inflammatory effects in the cells. Because** 1** and** 9** showed low cytotoxicity and regulated both PGE_2_ and NO production caused by inflammatory responses, they may hold promise as natural anti-inflammatory agents.

## Abbreviations

LPS: Lipopolysaccharide
iNOS: Inducible nitric oxide synthase
COX: Cyclooxygenase
IL: Interleukin
TNF-*α*: Tumor necrosis factor-*α*
ERK: Extracellular signal-regulated kinase
I*κ*B*α*: Nuclear factor of kappa light polypeptide gene enhancer in B-cells inhibitor-*α*
NF*κ*B: Nuclear factor-kappa B
TLRs: Toll-like receptors
LN: N^G^-monomethyl-L-arginine
NS: NS-398
TBS: *tert*-Butyldimethylsilyl
LiHMDS: Lithium bis(trimethylsilyl)amide
DMEM: Dulbecco's modified Eagle's medium
DMSO: Dimethyl sulfoxide
LDS: Lithium dodecyl sulfate
PVDF: Polyvinylidene fluoride
GAPDH: Glyceraldehyde-3-phosphate dehydrogenase
JNK: c-jun N-terminal kinase
p38MAPK: Mitogen-activated protein kinase p38
SD: Standard deviation
ANOVA: Analysis of variance
IM: Indomethacin
BIX: BIX02189
LY: LY294002
IC_50_: Half maximal inhibitory concentration.

## References

[1] McColl S. R., Hachicha M., Levasseur S., Neote K., Schall T. J. (1993). Uncoupling of early signal transduction events from effector function in human peripheral blood neutrophils in response to recombinant macrophage inflammatory proteins-1*α* and -1*β*. *Journal of Immunology*.

[2] Darville T., Jacobs R., Giroir B. (1993). The systemic inflammatory response syndrome (SIRS): immunology and potential immunotherapy. *Infection*.

[3] Toichi E., Hanada K., Hosokawa T. (1997). Age-related decline in humoral immunity caused by the selective loss of TH cells and decline in cellular immunity caused by the impaired migration of inflammatory cells without a loss of TDTH cells in SAMP1 mice. *Mechanisms of Ageing and Development*.

[4] Abd El-Aal A. A., Hassan M. A., Gawdat H. I., Ali M. A., Barakat M. (2016). Immunomodulatory impression of anti and pro-inflammatory cytokines in relation to humoral immunity in human scabies. *International Journal of Immunopathology and Pharmacology*.

[5] Devillier P. (2004). Synergic effects of anti-inflammatory drugs in asthma. *Archives de Pediatrie*.

[6] Bernardo A., Gasparini L., Ongini E., Minghetti L. (2006). Dynamic regulation of microglial functions by the non-steroidal anti-inflammatory drug NCX 2216: implications for chronic treatments of neurodegenerative diseases. *Neurobiology of Disease*.

[7] Okada T., Afendi F. M., Yamazaki M. (2016). Informatics framework of traditional Sino-Japanese medicine (Kampo) unveiled by factor analysis. *Journal of Natural Medicines*.

[8] Farkas O., Palócz O., Pászti-Gere E., Gálfi P. (2015). Polymethoxyflavone apigenin-trimethylether suppresses lps-induced inflammatory response in nontransformed porcine intestinal cell line IPEC-J2. *Oxidative Medicine and Cellular Longevity*.

[9] Hollman P. C. H., Katan M. B. (1997). Absorption, metabolism and health effects of dietary flavonoids in man. *Biomedicine and Pharmacotherapy*.

[10] De Groot H., Rauen U. (1998). Tissue injury by reactive oxygen species and the protective effects of flavonoids. *Fundamental and Clinical Pharmacology*.

[11] Gao D., Jin F., Liu H., Wang Y., Jiang Y. (2014). Metabonomic study on the antitumor effect of flavonoid derivative 3d in HepG2 cells and its action mechanism. *Talanta*.

[12] Friedman M. (2014). Antibacterial, antiviral, and antifungal properties of wines and winery byproducts in relation to their flavonoid content. *Journal of Agricultural and Food Chemistry*.

[13] Rodanant P., Khetkam P., Suksamrarn A., Kuvatanasuchati J. (2015). Coumarins and flavonoid from Murraya paniculata (L.) Jack: antibacterial and anti-inflammation activity. *Pakistan Journal of Pharmaceutical Sciences*.

[14] Zhong J., Colicino E., Lin X. (2015). Cardiac autonomic dysfunction: particulate air pollution effects are modulated by epigenetic immunoregulation of Toll-like receptor 2 and dietary flavonoid intake. *Journal of the American Heart Association*.

[15] Manabe K., Kyung K.-H., Shiratori S. (2015). Biocompatible slippery fluid-infused films composed of chitosan and alginate via layer-by-layer self-assembly and their antithrombogenicity. *ACS Applied Materials and Interfaces*.

[16] Rodgers R. J., Tschöp M. H., Wilding J. P. H. (2012). Anti-obesity drugs: past, present and future. *DMM Disease Models and Mechanisms*.

[17] Ninomiya M., Tanaka K., Tsuchida Y., Muto Y., Koketsu M., Watanabe K. (2011). Increased bioavailability of tricin-amino acid derivatives via a prodrug approach. *Journal of Medicinal Chemistry*.

[18] Ninomiya M., Nishida K., Tanaka K., Watanabe K., Koketsu M. (2013). Structure-activity relationship studies of 5,7-dihydroxyflavones as naturally occurring inhibitors of cell proliferation in human leukemia HL-60 cells. *Journal of Natural Medicines*.

[19] Kim M. H., Kim B. T., Min Y. K., Kim S. H. (2008). Profiling signalling pathways of the receptor activator of NF-*κ*B ligand-induced osteoclast formation in mouse monocyte cells, RAW264.7. *Amino Acids*.

[20] Rollo E. E., Laskin D. L., Denhardt D. T. (1996). Osteopontin inhibits nitric oxide production and cytotoxicity by activated RAW264.7 macrophages. *Journal of Leukocyte Biology*.

[21] Jang H.-S., Kim S. K., Han J.-B., Ahn H.-J., Bae H., Min B.-I. (2005). Effects of bee venom on the pro-inflammatory responses in RAW264.7 macrophage cell line. *Journal of Ethnopharmacology*.

[22] Nishina A., Kimura H., Sekiguchi A., Fukumoto R.-H., Nakajima S., Furukawa S. (2006). Lysophosphatidylethanolamine in *Grifola frondosa* as a neurotrophic activator via activation of MAPK. *Journal of Lipid Research*.

[23] Porter D. D., Porter H. G., Larsen A. E., Hadlow W. J. (1984). Immunoenzyme Western blotting, analysis of antibody specificity in Aleutian disease of mink, a parvovirus infection. *Journal of Virology*.

[24] Rodrigues A., Queiróz D. B. C., Honda L., Silva E. J. R., Hall S. H., Avellar M. C. W. (2008). Activation of Toll-Like Receptor 4 (TLR4) by in vivo and in vitro exposure of rat epididymis to lipopolysaccharide from Escherichia coli. *Biology of Reproduction*.

[25] Zhang T.-Z., Yang S.-H., Yao J.-F., Du J., Yan T.-H. (2015). Sangxingtang inhibits the inflammation of LPS-induced acute lung injury in mice by down-regulating the MAPK/NF-*κ*B pathway. *Chinese Journal of Natural Medicines*.

[26] Shalini V., Pushpan C. K., Sindhu G., Jayalekshmy A., Helen A. (2016). Tricin, flavonoid from Njavara reduces inflammatory responses in hPBMCs by modulating the p38MAPK and PI3K/Akt pathways and prevents inflammation associated endothelial dysfunction in HUVECs. *Immunobiology*.

[27] Park E. J., Park S. W., Kim H. J., Kwak J.-H., Lee D.-U., Chang K. C. (2014). Dehydrocostuslactone inhibits LPS-induced inflammation by p38MAPK-dependent induction of hemeoxygenase-1 in vitro and improves survival of mice in CLP-induced sepsis in vivo. *International Immunopharmacology*.

[28] Zha L., Chen J., Sun S. (2014). Soyasaponins can blunt inflammation by inhibiting the reactive oxygen species-mediated activation of PI3K/Akt/NF-kB pathway. *PLoS ONE*.

[29] Clark P. R., Jensen T. J., Kluger M. S. (2011). MEK5 is activated by shear stress, activates ERK5 and induces KLF4 to modulate TNF responses in human dermal microvascular endothelial cells. *Microcirculation*.

[30] Kültz D., Burg M. (1998). Evolution of osmotic stress signaling via MAP kinase cascades. *The Journal of Experimental Biology*.

[31] Wang X., Tournier C. (2006). Regulation of cellular functions by the ERK5 signalling pathway. *Cellular Signalling*.

[32] Obara Y., Yamauchi A., Takehara S. (2009). ERK5 activity is required for nerve growth factor-induced neurite outgrowth and stabilization of tyrosine hydroxylase in PC12 cells. *Journal of Biological Chemistry*.

[33] Sarközi R., Miller B., Pollack V. (2007). ERK1/2-driven and MKP-mediated inhibition of EGF-induced ERK5 signaling in human proximal tubular cells. *Journal of Cellular Physiology*.

[34] Korthuis R. J., Gute D. C. (2002). Anti-inflammatory actions of a micronized, purified flavonoid fraction in ischemia/reperfusion. *Advances in Experimental Medicine and Biology*.

[35] Kawai Y. (2014). *β*-Glucuronidase activity and mitochondrial dysfunction: the sites where flavonoid glucuronides act as anti-inflammatory agents. *Journal of Clinical Biochemistry and Nutrition*.

[36] Bandyopadhaya A., Das D., Chaudhuri K. (2009). Involvement of intracellular signaling cascades in inflammatory responses in human intestinal epithelial cells following Vibrio cholerae infection. *Molecular Immunology*.

[37] Thompson W. L., Van Eldik L. J. (2009). Inflammatory cytokines stimulate the chemokines CCL2/MCP-1 and CCL7/MCP-7 through NF*κ*B and MAPK dependent pathways in rat astrocytes. *Brain Research*.

[38] Kim S. H., Park J. G., Lee J. (2015). The dietary flavonoid kaempferol mediates anti-inflammatory responses via the src, syk, IRAK1, and IRAK4 molecular targets. *Mediators of Inflammation*.

[39] Naess P. A., Kirkebøen K. A., Christensen G., Kiil F. (1992). Inhibition of renal nitric oxide synthesis with NG-monomethyl-L-arginine and NG-nitro-L-arginine. *American Journal of Physiology—Renal Fluid and Electrolyte Physiology*.

[40] Amir S., De Blasio E., English A. M. (1991). NG-monomethyl-L-arginine co-injection attenuates the thermogenic and hyperthermic effects of E2 prostaglandin microinjection into the anterior hypothalamic preoptic area in rats. *Brain Research*.

[41] De Levai X., Dogné J.-M., Neven P. (1999). Effects of nimesulide and indometacin on COX-1 and COX-2: a comparative study. *Journal de Pharmacie de Belgique*.

[42] Peters M. W., Pursley J. R., Smith G. W. (2004). Inhibition of intrafollicular PGE2 synthesis and ovulation following ultrasound-mediated intrafollicular injection of the selective cyclooxygenase-2 inhibitor NS-398 in cattle. *Journal of Animal Science*.

[43] Nasrallah R., Laneuville O., Ferguson S., Hébert R. L. (2001). Effect of COX-2 inhibitor NS-398 on expression of PGE2 receptor subtypes in M-1 mouse CCD cells. *American Journal of Physiology—Renal Physiology*.

[44] Abarikwu S. O. (2014). Kolaviron, a natural flavonoid from the seeds of Garcinia kola, reduces LPS-induced inflammation in macrophages by combined inhibition of IL-6 secretion, and inflammatory transcription factors, ERK1/2, NF-*κ*B, p38, Akt, p-c-JUN and JNK. *Biochimica et Biophysica Acta—General Subjects*.

